# Investigation on Crude and High-Temperature Heated Coffee Oil by ATR-FTIR Spectroscopy along with Antioxidant and Antimicrobial Properties

**DOI:** 10.1371/journal.pone.0138080

**Published:** 2015-09-14

**Authors:** Diana Nicoleta Raba, Mariana-Atena Poiana, Aurica Breica Borozan, Marius Stef, Florina Radu, Mirela-Viorica Popa

**Affiliations:** 1 Banat’s University of Agricultural Sciences and Veterinary Medicine “King Michael I of Romania” from Timisoara, Faculty of Food Processing Technology, Calea Aradului 119, 300645 Timisoara, Romania; 2 Banat’s University of Agricultural Sciences and Veterinary Medicine “King Michael I of Romania” from Timisoara, Faculty of Horticulture and Forestry, Calea Aradului 119, 300645 Timisoara, Romania; 3 West University of Timisoara, Faculty of Physics, Bd. Vasile Parvan 4, 300223 Timisoara, Romania; University of Sassari, ITALY

## Abstract

The coffee oil has a promising potential to be used in food industry, but an efficient use, especially in products that required high-temperature heating, depends on its chemical composition and the changes induced by processing. Since there is little information on this topic, the aim of our study was to investigate the crude green and roasted coffee oil (GCO, RCO) and heated (HGCO, HRCO) for 1 h at 200°C, by Fourier Transform Infrared (FTIR) spectroscopy and in terms of antioxidant and antimicrobial properties. The results of FTIR spectroscopy revealed that no statistically significant differences (one-way ANOVA, p>0.05) in the oxidative status of GCO and RCO were found. The coffee oils heating induced significant spectral changes in the regions 3100–3600 cm^–1^, 2800–3050 cm^–1^ and 1680–1780 cm^–1^ proved by the differences in the absorbance ratios A 3009 cm^−1^/A 2922 cm^−1^, A 3009 cm^−1^/A 2853 cm^−1^, A 3009 cm^−1^/A 1744 cm^−1^, A 1744 cm^−1^/A 2922 cm^−1^. These alterations were related to the reduction of the unsaturation degree due to primary and secondary oxidation processes of the lipid fraction. The radical scavenging ability of oils investigated by 2,2-diphenyl-1-picrylhydrazyl (DPPH) assay revealed that the IC50 value of GCO was significantly lower than of RCO (p<0.05). The IC50 values of crude coffee oils were lower than those of heated samples. The antioxidant activity of oils was attributed to both antioxidant compounds with free-radical scavenging capacity and to lipids oxidation products generated by heating. In the first 6 h of incubation, the inhibitory activity of crude oils against *E*. *coli* and *E*. *faecalis* was not significantly different to the control (p>0.05). Also, HGCO and HRCO showed significantly different inhibitory potential related to the control (p<0.05). The heating induced statistically significant decreases in the effectiveness of coffee oils against the tested bacteria. GCO proved to be the most effective among investigated coffee oils against the tested bacteria.

## Introduction

The oil extracted from green and roasted coffee beans is a naturally rich source of valuable bioactive phytochemicals, most of them showing promising significance in nutrition (because of its antioxidant activity), in the pharmaceutical industry (dry and cracked skin, eczema, psoriasis and other skin-related diseases) and in cosmetics (due to its balanced composition in fatty acids) [1–4]. An efficient use of coffee oil in food industry greatly depends not only on its chemical composition but also on the changes induced by processing. However, there is little information about the differences among green and roasted coffee oil as well as how the exposure to high temperature influences the oxidative stability, the antioxidant and the antimicrobial activity of coffee oil, respectively. This was the main reason that drove us towards the systematic study of chemical and functional properties of crude and heat-treated green and roasted coffee oil. The lipid fraction of coffee beans represents 7–17% on dry basis, depending on the coffee species: Arabica coffee has around 5% more than Robusta [5,6]. The saponifiable fraction of lipids extracted from green coffee mainly consists of triacylglycerols (about 75%), phospholipids and kaurane esterified diterpenes [4,7]. The main fatty acids of green coffee oil are palmitic and linoleic acids [3,4]. The large percentage of unsaponifiable fraction contains sterols, tocopherols, free diterpenes, waxes and various other minor components whose biological properties including antioxidant, antimicrobial and antiproliferative activity have been previously reported [5,8]. Among the different diterpenes found in unsaponifiable fraction, cafestol and kahweol are of interest due to their anti-inflammatory properties and potential anticarcinogenic effects [2,9]. The roasting of green coffee beans induces many chemical changes such as the formation of volatile compounds [10] and Maillard reaction products. These products are responsible for the intense aroma of roasted coffee [2,11], but the coffee roasting has no effect on the oxidation level of the lipid fraction [12,13].

Nowadays, there is a great interest in using the RCO as ingredient in a wide range of food formulations because it presents peculiar flavoring and nutraceutical characteristics being able to enhance the health-protecting capacity of these products [1,2]. Therefore, it could be used for increasing the aromatic potential of instant coffee and coffee beverages, as well as a flavoring ingredient for ice creams, candies, ready-to-drink beverages, cakes and puddings [1,14]. GCO is used as emollient in the cosmetics industry due to its special composition balanced in fatty acids as well as to its ability to block harmful sunlight [3,15]. Also, because of its antioxidant properties, GCO is used in pharmaceuticals, in the formulation of creams and ointments designed to improve the skin condition in the anti-aging treatments and in various skin diseases [4,16,17].

Data reported by Wagemaker *et al*. [4] revealed that GCO showed low antioxidant activity and it did not exhibit antimicrobial activity against some bacteria and *Candida albicans*. A lot of work has been done in attempting to control pathogenic microorganisms responsible for food-borne diseases, occurring by the consumption of contaminated foods [18,19]. It is widely known that *Escherichia coli* and *Enterococcus faecalis* are among the common pathogens responsible for food-borne illnesses [20], but there are no literature data regarding the activity of coffee oil against these bacteria.

In the light of the increasing interest in the antioxidant and antimicrobial properties of food ingredients, coffee oil could be used to enhance the preservation of a food formulation. An interesting application of coffee oil could be its use in pastry products that need thermal processing up to 60 minutes at high temperatures in the range 180–200°C. Nowadays, there are no available data regarding the alterations induced in coffee oil quality in response to thermal stress. Lipid oxidation is the major degradative process, based on a free radical chain mechanism, occurring during heat treatment of food containing lipid molecules with polyunsaturation. The lipid oxidation products lead to the loss of nutritional value and safety of food, resulting in undesirable nutritional effects on consumers [21]. It is well known that in the early stage of the thermally lipid oxidation process, free radicals including alkyl (R∙), alkoxyl (RO∙), and peroxyl radicals (ROO∙) are formed [22,23]. Recent studies confirm that 2,2-diphenyl-1-picrylhydrazyl (DPPH) free radicals can be used for the determination of the degree of oxidation and antioxidant effectiveness of free radical scavengers in oils [24]. Thus, the DPPH modified method could be used as an indication of oxidative stability and antioxidant capacity of these products.

The studies carried out in the last decades have proven the feasibility of FTIR spectroscopy to characterize edible oils, but also to assess the thermo-oxidative changes induced in edible oils undergoing thermal stress by using vibrations of various chemical groups at specific wavelengths in the mid-infrared region of the spectrum [25,26,27]. Moreover, other authors suggested the use of some ratios of the absorbance of specific bands recorded in the FTIR spectra to reveal the change induced in edible oils by thermal stress [28,29]. Considering all the information mentioned above, our purpose in this paper is to address two important practical questions: (1) What are the differences among GCO and RCO evaluated by Attenuated Total Reflectance–Fourier Transform Infrared (ATR-FTIR) spectroscopy as well as the differences in their DPPH radical scavenging activity and antimicrobial properties? (2) How does the high-temperature heating influence the crude coffee in terms of oxidative stability, antioxidant and antimicrobial properties? To this end, GCO and RCO were extracted from green and roasted Arabica coffee beans in a Soxhlet extractor apparatus, and then both crude coffee oils were continuously heated to 200°C over 1 h. Further, crude and heated coffee oils were investigated by ATR-FTIR spectroscopy and in terms of analytical constants, antioxidant activity by DPPH assay and antimicrobial properties. The results of this study are important because they provide subsidiary information about the properties of GCO and RCO, as well as their behavior during high-temperature heat treatment, with reference to their potential to be further used as ingredients in food industry.

## Materials and Methods

### Samples

For obtaining the crude coffee oil in order to be further used in analytical purposes for detailed analysis, direct solvent extraction was necessary [8]. In this study were used green and roasted Arabica Typica coffee beans originated from Guaxupe (Brazil), purchased from a Romanian coffee distributor (SC Global Trade SRL, 24 Constructorului Street, Cornetu—Ilfov County, geographical coordonates: 44°21′01″N; 25°57′05″E), lot number 922/1456. Prior extraction, coffee beans were ground using a grinder (Grindomix Retsch GM 2000) and passed through a 60 mesh sieve. Crude oil from ground green and roasted coffee was extracted in a Soxhlet extractor apparatus by repeated washing (15 percolations) with petroleum ether (1:3, w/v) for 3 h.

### Heat treatment of coffee oil samples

20±0.5 g of crude coffee oil samples were weighed in Pyrex Petri dishes (10 cm inner diameter) and placed without their lids in a forced air oven (Froilabo AC60/France, 1000 W) and continuously exposed to heat, over 1 h at 200±1°C. The temperature of oil samples subjected to heating was monitored by a calibrated chromel-alumel thermocouple (HI 935009, Hanna Instruments). After thermal treatment, the heated green and roasted coffee oils (HGCO and HRCO) were taken out of the oven, covered, allowed to cool at room temperature, poured into glass bottles and then, stored in dark, in refrigeration conditions at 4–6°C until analysis. Heating treatment was carried out in duplicate. The coffee oil samples were kept at room temperature (25°C) for 24 h prior to FTIR measurements.

### Evaluation of analytical constants of coffee oil

Crude and heat-treated coffee oils were investigated in terms of free fatty acids value, unsaponifiable matter, peroxide value, iodine value and saponification value according to standard methods for oils analysis [30].

### ATR-FTIR spectra acquisition

IR spectra were measured at room temperature in the range 400–4000 cm^−1^ on Thermo Nicolet Nexus 470 FTIR Spectrometer from Thermo Nicolet Corp., Madison, WI, USA equipped with attenuated total reflectance (ATR) accessory. In order to obtain a good signal-to-noise ratio DTGS detector and XT-KBr beamspliter was used. FTIR spectra were subtracted against the background of air spectrum. Background scan and coffee oil samples were sequentially measured with a spectral resolution of 4 cm^−1^ (0.482 cm^−1^ data spacing). A background of new reference air spectrum was taken after every scan of coffee oil sample. An aliquot of 1 ml of the oil sample in a thin film was used for FTIR spectra recording. These spectra were registered at room temperature, as absorbance values at each data point in triplicate. The baseline correction was applied to all spectra using fixed points method.

### DPPH Radical-scavenging Assay

Radical scavenging activity of coffee oils was evaluated by the reduction of DPPH in toluene according to the method described by Ramadan *et al*. [31]. For this purpose, 1 ml solution of each coffee oil in toluene (at final concentrations of 5, 10, 15, 20 and 25 mg/ml) was mixed with 4 ml freshly prepared toluene solution of DPPH 0.004% (w/v), and the obtained mixture was vortexed for 10 s and then incubated for 30 minutes at ambient temperature (20°C). Also, 1 ml toluene solutions of BHT (as standard), at concentrations of 50, 100, 200, 300 and 400 μg/ml, were mixed with 4 ml toluene solution of DPPH and prepared as previously, in the case of coffee oil samples. The decrease in the absorption at 517 nm was measured in 1-cm glass cells against a blank of pure toluene without DPPH using a UV–VIS spectrophotometer (SPECORD 205, Analytic Jena). The capability to scavenge the DPPH radicals was calculated as a percentage of DPPH discoloration, on the basis of Eq (1):

$$\text{DPPH inhibition}\;(\%)=\frac{{\text{A}}_{\text{C}}\;\text{-}\;{\text{A}}_{\text{S}}}{{\text{A}}_{\text{C}}}\times 100$$ (1)

Where A_C_ represents the absorbance of the DPPH solution (control) and A_S_ is the absorbance in the presence of sample (toluene solution of coffee oils and BHT, respectively). The IC50 value, defined as the sample concentration that inhibits the formation of DPPH radicals by 50%, was determined from the regression equation obtained by plotting DPPH inhibition percentage against sample concentration.

### Antimicrobial assay

The test was performed to evaluate *in vitro* antimicrobial activity of crude and heated coffee oil samples against some common food-related bacteria using the Kirby-Bauer method as recommended by the National Committee for Clinical Laboratory Standard [32].

### Bacterial culture

Gram-positive bacteria: *Enterococcus faecalis* (ATCC 29212) and *Staphylococcus aureus* (ATCC 25923) and Gram-negative bacteria: *Salmonella* (ATTC 14028), *Shigella flexneri* (ATCC 12022) and *Escherichia coli* (ATCC 25922) were used as reference microbial strains for the antimicrobial assays. These tested bacteria were inoculated on broth media for 24 h at 37°C.

### Disc Diffusion Test

The antimicrobial activity was evaluated by the disc diffusion method according to CLSI [32]. Briefly, the Mueller-Hinton agar media (3 mm layer) was introduced into Petri dishes (8 cm inner diameter) and incubated in a thermostat (30 minutes at 37°C) in order to dry the media surface. Afterwards, the Mueller-Hinton agar media was inoculated with a suspension of the tested microorganisms (10^6^ cells/ml), previously grown on broth media. The paper discs, 6 mm in diameter (Whatman no. 1), were equidistantly disposed on the surface of the Mueller-Hinton agar media. Further, 10 μl of oil samples were added on the test discs using a micropipette. Ampicillin (10 μg/disc, HiMedia Laboratories Pvt. Limited, Mumbai, India) used as control against *E*. *faecalis*, *Salmonella*, *S*. *flexneri*, *S*. *aureus* and Cefotaxime (30 μg/disc, Oxoid Laboratories, UK) as control against *E*. *coli*, were applied on the Mueller-Hinton media. The microbial plates were sealed with sterile Parafilm and then incubated at 37°C for 6 h and 18 h, respectively. All diffusion tests were carried out in three repetitions. The antimicrobial activity was expressed as the mean of inhibition zone diameter (D, mm) measured after incubation to an accuracy of 1 mm.

### Stasistical Analysis

All determinations were performed in triplicates and the results were reported as means ± standard deviation (SD). Statistical data processing was carried out by one-way analysis of variance (ANOVA) to establish statistical significance in the differences observed. Computations Tukey post-hoc means comparisons was included to appreciate the significance of the recorded differences. The statistical analysis was done using the Statistical Analysis System, SAS (Software Version 8.1; SAS Institute, Inc., Cary, NC) [33]. The differences were considered statistically significant at a probability value p<0.05.

## Results and Discussion

### Assessing the analytical data of coffee oil

The analytical data of coffee oil samples after extraction from green and roasted coffee beans and in response to heating for 1 h at 200°C is shown in Table 1. The statistical significance of the differences observed among coffee oil samples were appreciated after data processing by one-way ANOVA test.

**Table 1:** Analytical constants of coffee oil.

Analitical constants	GCO	HGCO	RCO	HRCO
Unsaponifiables (%)	8.09±0.39 ^a^	6.97±0.35 ^b^	6.34±0.29^b^	5.53±0.25^c^
Free fatty acids (%, as oleic acid)	1.91±0.085^a^	3.32±0.15^c^	2.64±0.11^b^	4.08±0.18^d^
Iodine value (g I_2_/100 g)	88.91±2.47^a^	80.75±1.73^b^	86.24±1.88^a^	79.04±1.52^b^
Perodixe value (meq O_2_/kg)	3.20±0.15^a^	8.17±0.37 ^c^	5.14±0.22^b^	14.81±0.65^d^
Saponification value (mg KOH/g)	185.86±3.1^a^	193.21±4.08^b^	179.29±3.63^a^	184.32±3.99^a^

Values within the same row sharing different superscripts (a-d) are significantly different from each other (one-way ANOVA, p<0.05).

To our knowledge, the study of thermo-oxidation effects induced in response to heating at high temperature on analytical characteristics of green and roasted coffee oil has not yet been done. Our results revealed the relatively large content of unsaponifiable fraction found in both heated and untreated coffee oil samples. Statistically significant differences (p<0.05) in unsaponifiable matter were registered between GCO and thermal treated oils. These values are consistent to those obtained by Wagemaker *et al*. [3] and Ravindranath *et al*. [34] who reported significant amounts of unsaponifiable matter which greatly varies in coffee beans and may reach levels of up to 13.5%. The unsaponifiable matter would be responsible for moisture binding in coffee oil [3]. This fact could affect the further behavior of coffee oil during high-temperature heating, promoting the hydrolysis of triglycerides.

The free fatty acids values in untreated coffee oil samples measure the extent of the triglycerides decomposition by lipase action in green and roasted coffee beans. Our results reveal a lower value of free fatty acids in GCO (1.91% as oleic acid) than in RCO (2.64% as oleic acid). The obtained results are in agreement with the results reported on this topic by Hartman *et al*. [35]. The free fatty acids content increased by heating, as a result of increasing deterioration, reaching the value of 3.32% for HGCO, respectively 4.08% for HRCO. Statistical analysis, have confirmed that the high-temperature heating led to a significant increase of free fatty acids content of coffee oils (p<0.05).

The iodine value provides information about the coffee oil samples unsaturation. The recorded values were similar for both GCO and RCO and situated in the range 85–99 g I_2_/100 g, available in the literature for coffee oil [36]. Statistically no significant difference was registered between crude coffee oils (p>0.05). The slightly lower value found for RCO may be attributed to the starting of oxidation process to the double bonds belonging to unsaturated fatty acids of oil samples. Statistical test revealed that the iodine value significantly decreased (p<0.05) after the heating of both crude coffee oil samples, as a result of the decrease in the degree of unsaturation. This decrease is an indication of the reduction occurring in unsaturated fatty acids due to thermal oxidation.

The saponification value of coffee oil offers information on the character of the fatty acids of the investigated sample. This index is considered as a measure of the average molecular weight, or chain length, of all fatty acids existing in coffee oil sample. As shown in Table 1, GCO had higher saponification values than RCO. Statistically insignificant differences between crude coffee oils were recorded (p>0.05). The heat treatment led to higher saponification values of both coffee oil samples. Statistical analysis showed a significant difference between GCO and HGCO (p<0.05). Peroxide value is one of the most frequently quality parameters evaluated during oil production, storage and marketing. The peroxide value provides information about the degree of coffee oil oxidation and measures the amount of total peroxides, as primary oxidation products. The data presented in Table 1 show that none of the crude coffee oil samples were above the limit of 10 meq O_2_/kg for edible oils. The increases noticed in the peroxide value in response to heating, revealed the formation of hydroperoxides as primary oxidation compounds. The differences recorded in the peroxide values of crude and heated coffee oil samples were statistically significant (one-way ANOVA, p<0.05), Table 1. The highest value was recorded in HRCO (14.81 meq O_2_/kg).

### Assessing the ATR-FTIR spectra of coffee oil

**Fig 1:**
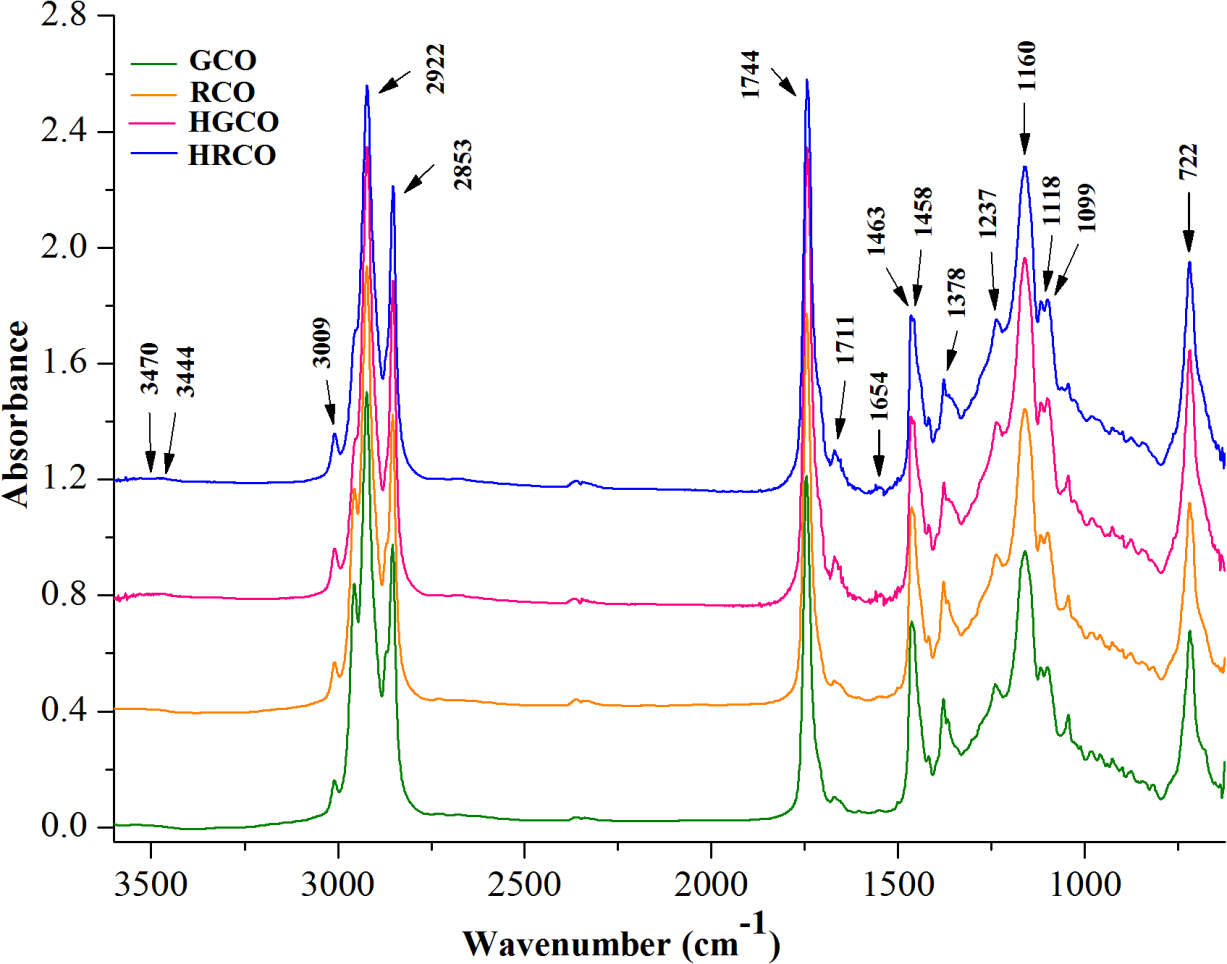
Comparative ATR-FTIR spectra of coffee oil.

ATR–FTIR spectroscopy was used for the structural differentiation of crude coffee oils as well as to follow the changes induced by heating at 200°C for 1h. The spectra depicted in Fig 1 show characteristic bands whose frequencies and intensities can clearly reveal the functional groups of investigated oil samples.

The absorption bands of the infrared spectra were assigned to responsible functional groups on the basis of available literature data about edible oils (Table 2).

**Table 2:** Analytical evaluation of infrared spectra of coffee oil.

Wavenumbers	Responsible functional groups
3470 cm^–1^	Overtone of the glyceride ester carbonyl absorption (C = O) [21,29].
3444 cm^–1^	O-H stretching vibration of hydroperoxides [21].
3009 cm^–1^	C–H stretching symmetric vibration of the *cis* double bonds,
	(HC = CH) [26,29].
2922 and 2853 cm^–1^	Asymmetric and symmetric stretching vibration of C–H bonds of
	aliphatic CH_2_ group of the fatty acid backbone [37].
shoulders at 2956	Symmetric and asymmetric stretching vibration of C–H bonds of
and 2871 cm^–1^	aliphatic CH_3_ group [26,37].
1744 cm^–1^	Stretching vibration of ester carbonyl functional groups of
	triglycerides (O-C = O) [26].
weak shoulder at	Stretching vibration of free fatty acid carbonyl group (C = O) [29,37].
1711 cm^–1^	
1654 cm^–1^	C = C stretching vibration *cis*-olefins (*cis* RHC = CHR) [25,26].
1463 and 1458 cm^–1^	Bending vibration of C–H of CH_2_ and CH_3_ aliphatic group [29].
1418 cm^–1^	Rocking vibration of C–H bonds of *cis-*disubstituted olefins [26,29].
1397 cm^–1^	Bending in plane vibrations of C-H bonds of *cis*-olefinic group
	[25,26,29].
1378 cm^–1^	Bending symmetric vibration of C–H bonds of CH_2_ group [26].
1237 and 1160 cm^–1^	Stretching and rocking vibration of C–O ester group, CH_2_ [29].
1118 and 1099 cm^–1^	Stretching vibration of C–O ester group [37].
966 cm^−1^	Out-of-plane bending vibration of *trans*–HC = CH− group of
	disubstituted olefins [37].
914 cm^–1^	Out-of-plane bending vibration of *cis–*HC = CH–group [37].
722 cm^–1^	Overlapping of aliphatic CH_2_ rocking vibration and the out of plane
	vibration of *cis*-disubstituted olefins [26, 29].

**Fig 2:**
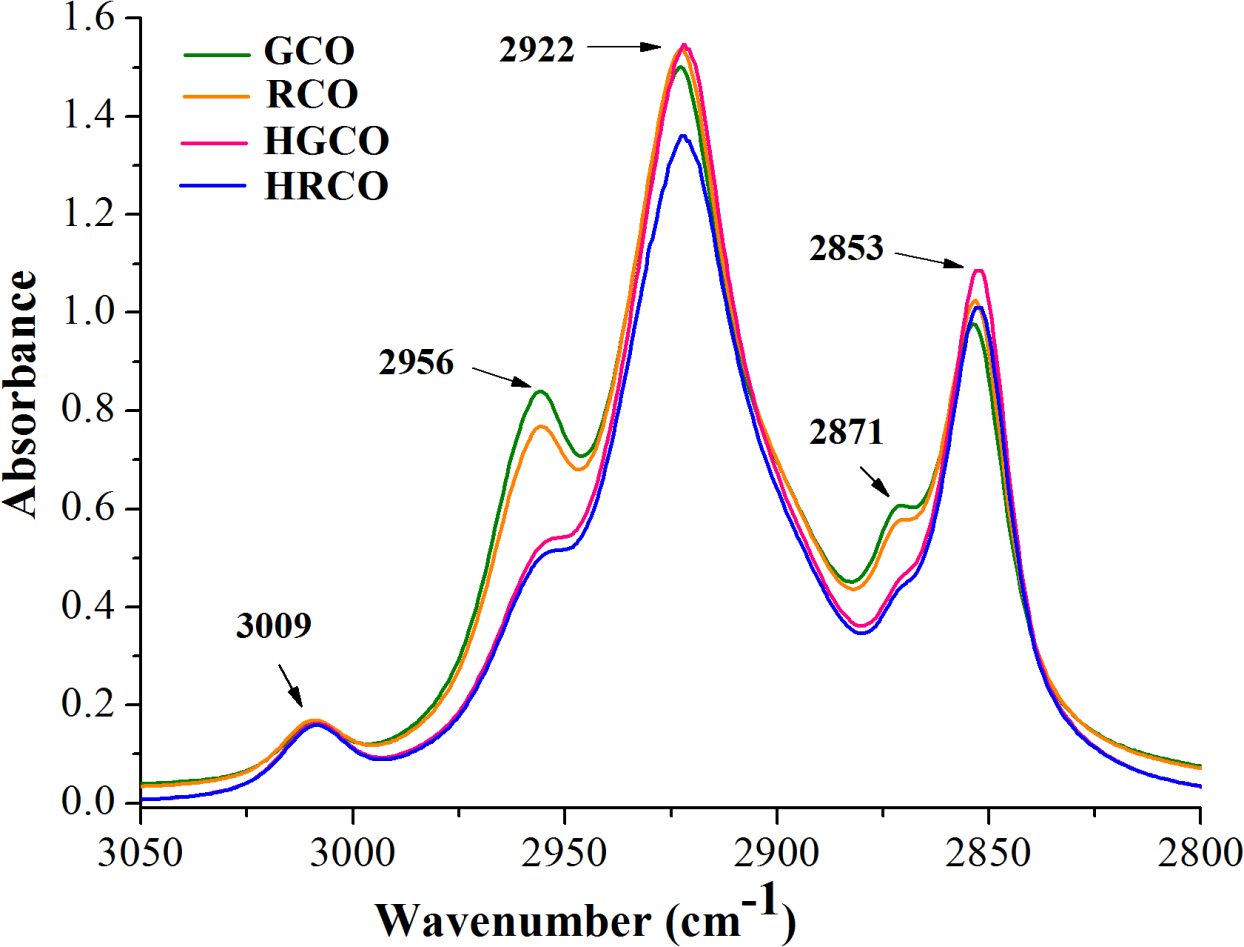
The region of hydrogen’s stretching vibrations of coffee oil infrared spectra.

**Fig 3:**
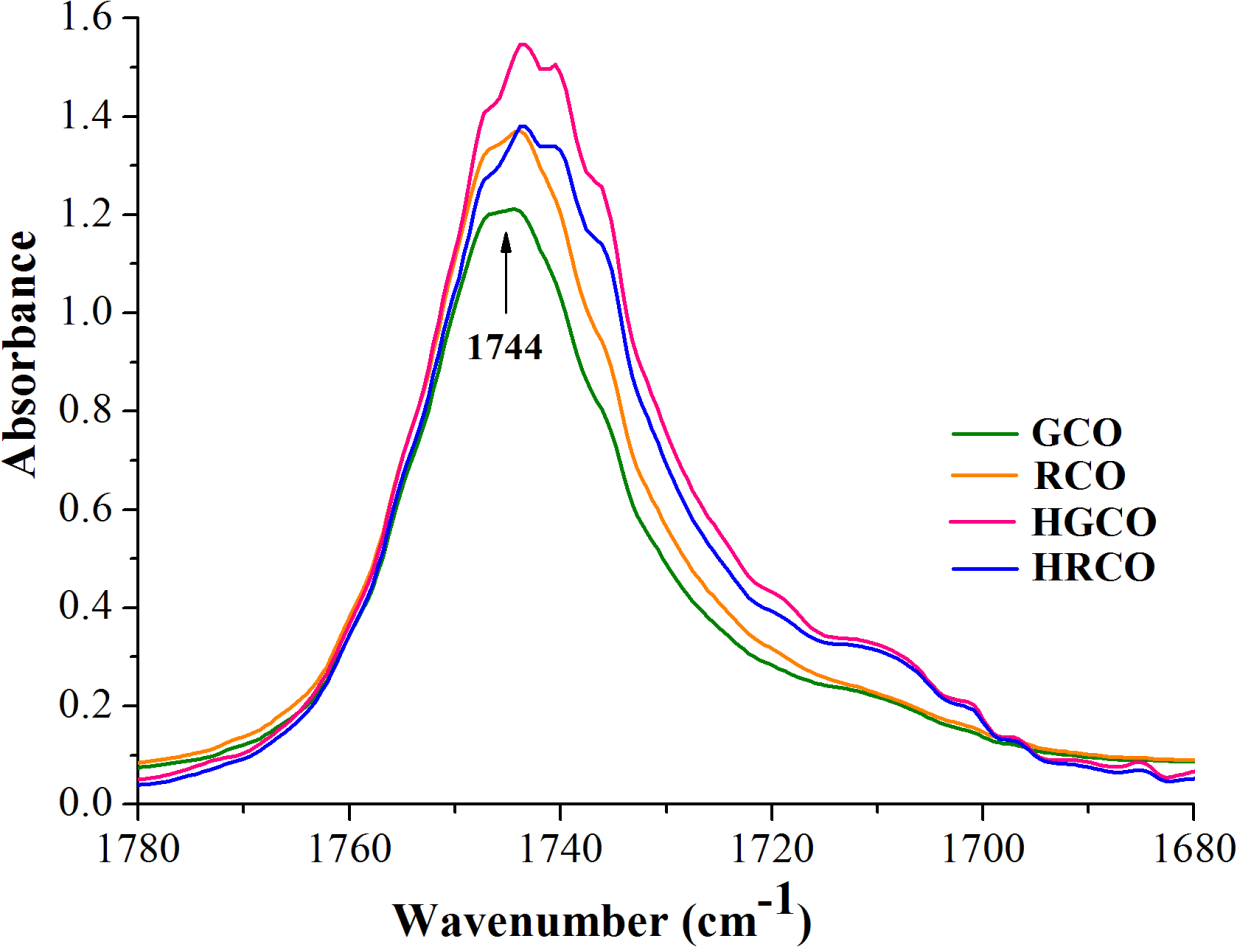
The region of carbonyl stretching vibrations of coffee oil infrared spectra.

The visual examination of ATR-FTIR spectra of both crude and heated coffee oil samples revealed that there are no recorded appreciable differences in their spectral features, apart from changes in the intensity of some absorption bands. Also, it can be noted that there were no registered shifts in the exact position of the recorded bands. At a closer look to the recorded spectra, the most relevant changes were noticed in the following regions, as follows: the OH stretching region of hydroxylic groups between 3100 and 3600 cm^–1^ (Fig 1), the region of hydrogen’s stretching vibrations between 2800 and 3050 cm^–1^ (Fig 2) and the region of carbonyl stretching vibrations between 1680 and 1780 cm^-1^ (Fig 3). The spectral changes detected in response to heating in these regions provided information about the differences in the oxidative state of the lipid fraction of coffee oil samples.

By comparing the spectra of crude and heated coffee oils in the region between 3600 and 3100 cm^–1^ (Fig 1), there is a very small band around 3470 cm^–1^, in crude oils, associated with the overtone of the glyceride ester carbonyl absorption [21]. In the coffee oil samples that underwent thermo-oxidative processes, this band becomes more intense and broader than in crude oils, with a maximum around 3444 cm^–1^ due to the presence of hydroperoxides. The band of hydroperoxides was too small to be detected in crude coffee oils because the hydroperoxides amount was still low in these samples. The concentration of the hydroperoxides increased as the oxidation progressed in response to high-temperature heating and its absorption band also increased. In agreement with the study performed by Guillen and Cabo [21] on heated oil samples, the hydroperoxides led to the formation of a broad band that overlaps with the band assigned to the overtone of the glyceride ester groups. The region depicted in Fig 2 is dominated by a series of aliphatic vibrations around 3000 cm^–1^, due to the large amount of CH_2_, and CH_3_ groups present in the fatty acids of oil samples. The stretching vibration of CH bonds lead to high absorptions registered at 2922 and 2853 cm^–1^ with two shoulders at 2956 and 2871 cm^–1^ [26,37]. Also, unsaturated fatty acids present in coffee oil gave a small peak at 3009 cm^–1^ (Fig 2), characteristic to the C–H stretching vibration of *cis* double bonds [26,29]. In the region shown in Fig 3 it can be seen the intense carbonyl peak at 1744 cm^–1^, due to the ester bonds between glycerol and fatty acids [37]. The careful examination of GCO and RCO infrared spectra revealed that irrespective of the oil type, there were no important modifications recorded in the intensity of the band registered at 3009 cm^–1^. Concerning the impact of heat treatment on coffee oil samples, it can be observed that the changes in dominant spectral features are associated with the coffee oil type, green respectively roasted. Under oxidative conditions, the band at 3009 cm^−1^ assigned to *cis* double bonds in the aliphatic chain begins to slightly decrease for both green and roasted coffee oil samples. Thus, it can be assumed that, during the heating of oil samples, the *cis* double bonds are consumed due to the isomerisation to *trans* groups and/or to their breakdown to produce secondary oxidation products. The differences in the spectra of heated oils show that, as oxidation proceeds, hydroperoxides are formed along with secondary oxidation products [38].

The vibration of the ester carbonyl group C = O of the triglycerides includes the spectral region between 1720 and 1750 cm^−1^. The carbonyl band consists of two components: a sharp peak appeared at 1744 cm^−1^ and another broad around 1728 cm^−1^ [39]. In terms of the changes occurring in the C = O region, Fig 3 reveals an increase in the intensity of the band recorded at 1744 cm^–1^ and also a slight widening of this band for RCO.

The spectral features of RCO were not significantly different from those of GCO, indicating that the oxidative stability of lipid fraction was not substantially affected by coffee beans roasting. This finding is supported also by the peroxide values of GCO and RCO, Table 1. As it was previously reported by Anese *et al*. [12] and Vila *et al*. [13], the roasting of coffee beans has no influence on the oxidation level of the lipid fraction, but the main changes, associated to the hydrolysis of triacylglicerols and to the oxidation of free fatty acids, occur during storage of roasted coffee beans [40]. The stability of lipid fraction of RCO could be explained by the presence of lipid-soluble colored Maillard reaction products formed during roasting, with antioxidant properties [41,42,43]. It is interesting to notice a more pronounced widening of the band recorded at 1744 cm^–1^ for HGCO and HRCO as a result of thermo-oxidation. Also, an obvious increase in the intensity of the band at 1744 cm^−1^ for HGCO is illustrated in Fig 3. These changes are associated with the degradation of hydroperoxides and the formation of saturated aldehydes, or other secondary oxidation products such as alcohol, ketones, acids and esters [21,26,29]. Aldehyde compounds exhibit bands in the region 1680–1730 cm^–1^ due to the high absorptivity of their carbonyl groups, which overlap with the strong band of the ester carbonyl functional group of the triglycerides recorded at 1744 cm^–1^. This fact induces a broadening of the band at 1744 cm^−1^ to lower frequencies, as new carbonyl groups are formed [26,29]. The differences induced in the C = O region in response to heating of GCO were more significant than those registered by RCO heating. This finding might be due to the protective effects of Maillard products from RCO against the thermo-oxidation [43]. Oxidation and other thermal alterations such as *cis*-*trans* isomerisation, cyclization and polymerization may take place in the oil samples during heating due to the presence of air and exposure to high temperature [44]. Additionally, the thermal oxidative cleavage of triglycerides, as a result of thermal stress exposure, leads to the formation of free fatty acids in the oil [45]. The releasing of the free fatty acids during heat exposure is proved by the increasing in the intensity of the band at 1711 cm^–1^ assigned to the stretching vibration of free fatty acid carbonyl group.

The relation between the absorbance ratios and the degree of unsaturation of oils was investigated by several authors. Their results proved a close relationship between the degree of unsaturation of vegetable oils expressed as iodine value and the ratios A 3009 cm^−1^/A 2922 cm^−1^ (RI), A 3009 cm^−1^/A 2853 cm^−1^ (RII) and A 3009 cm^−1^/A 1744 cm^−1^ (RIII) [28,29]. These ratios were considered as a reliable measure of the changes in the degree of the oil samples unsaturation in response to heat treatment. Additionally, the absorbance ratio A 1744 cm^−1^/A 2922 cm^−1^ (RIV) was analyzed according to Moharam and Abbas [29] for assessing the thermo-oxidative alterations. Table 3 shows the changes induced in the absorbance ratios by heat exposure of coffee oil samples. The statistical significance of the differences recorded for ratios RI-IV among coffee oil samples was established on the basis of one-way ANOVA test.

**Table 3:** The absorbance ratios RI-IV of coffee oil.

Ratio	GCO	HGCO	RCO	HRCO
**RI**	0.1076±0.005^a^	0.1051±0.003^a^	0.1102±0.004^a^	0.1169±0.004^b^
**RII**	0.1654±0.007^a^	0.1495±0.005^b^	0.1654±0.006^a^	0.1574±0.005^a^
**RIII**	0.1333±0.008^a^	0.1051±0.005^c^	0.1235±0.007^a^	0.1152±0.006^b^
**RIV**	0.8072±0.031^a^	1.0000±0.036^b^	0.8924±0.037^a^	1.0147±0.051^b^

Values within the same row sharing different superscripts (a-c) are significantly different from each other (one-way ANOVA, p<0.05).

The first thing we could notice about these data is that, there were no recorded statistically significant differences (one-way ANOVA, p>0.05) between the ratios RI-RIV of GCO and RCO. This finding support the results according to which the roasting did not induce oxidative alterations in the lipid fraction of coffee beans [12,13]. Another finding that emerges from these data analysis is that the high-temperature exposure of coffee oils induced some changes in the absorbance ratios RI-IV, but the differences were not statistically significant in all cases, Table 3. The ratios RI-RIII of GCO and RCO revealed decreases induced by high-temperature heating. The only exception has been observed for ratio RI of HRCO that recorded a statistically significant increase (p<0.05) when RCO was exposed to the heat treatment. This fact is a strong evidence for increasing in the degree of unsaturation that could be explained by the formation of primary oxidation products that contain both double bonds in *cis* form as well as conjugated double bonds [29,46].

It must be mentioned that, the decreases in the values of ratio RIII resulted by both crude coffee oils heating, as well as the changes induced in the RII value by high-temperature exposure of GCO were statistically significant different (p<0.05). The decrease in the absorbance ratios after heat exposure was related to the decrease in the degree of unsaturation, attributed to the reduction in the unsaturated fatty acids content (18:2 and 18:3) [38,46]. The decrease in the degree of unsaturation, may be due to the fact that, the rate of formation of primary oxidation products was lower than the rate of their degradation. The degradation of primary oxidation products to secondary oxidation products during heat exposure led to the decreasing of the degree of unsaturation as a consequence of the reduction in the *cis* double bonds. Our results are in agreement with those previously reported by Moreno *et al*. [47].

In addition to the mentioned decomposition of primary oxidation products, the decrease of these ratios could also be due to the scavenging of primary oxidation products like free radicals that contains *cis* double bonds by antioxidant compounds such as tocopherols found in coffee oil [29].

Regarding the RIV values, it can be seen an increase of these ratio in response to heat exposure of crude oils. The differences recorded in the RIV values of crude and heated oil samples were statistically significant (p<0.05) revealing the new carbonyl groups due to the formation of secondary oxidation products such as aldehydes, alcohol, ketones, acids and esters. The information provided by RIV supports the results obtained by analyzing of the ratios RI-III highlighting the oxidative changes attributed to the primary and secondary oxidation processes of the lipid fraction of coffee oil samples undergoing thermal stress.

These results attest to the value of FTIR spectroscopy, as a simple and efficient means, to characterize the crude coffee oils and also to follow the complex oxidative changes induced by high-temperature heating.

### Assessing the antioxidant activity of coffee oil

**Fig 4:**
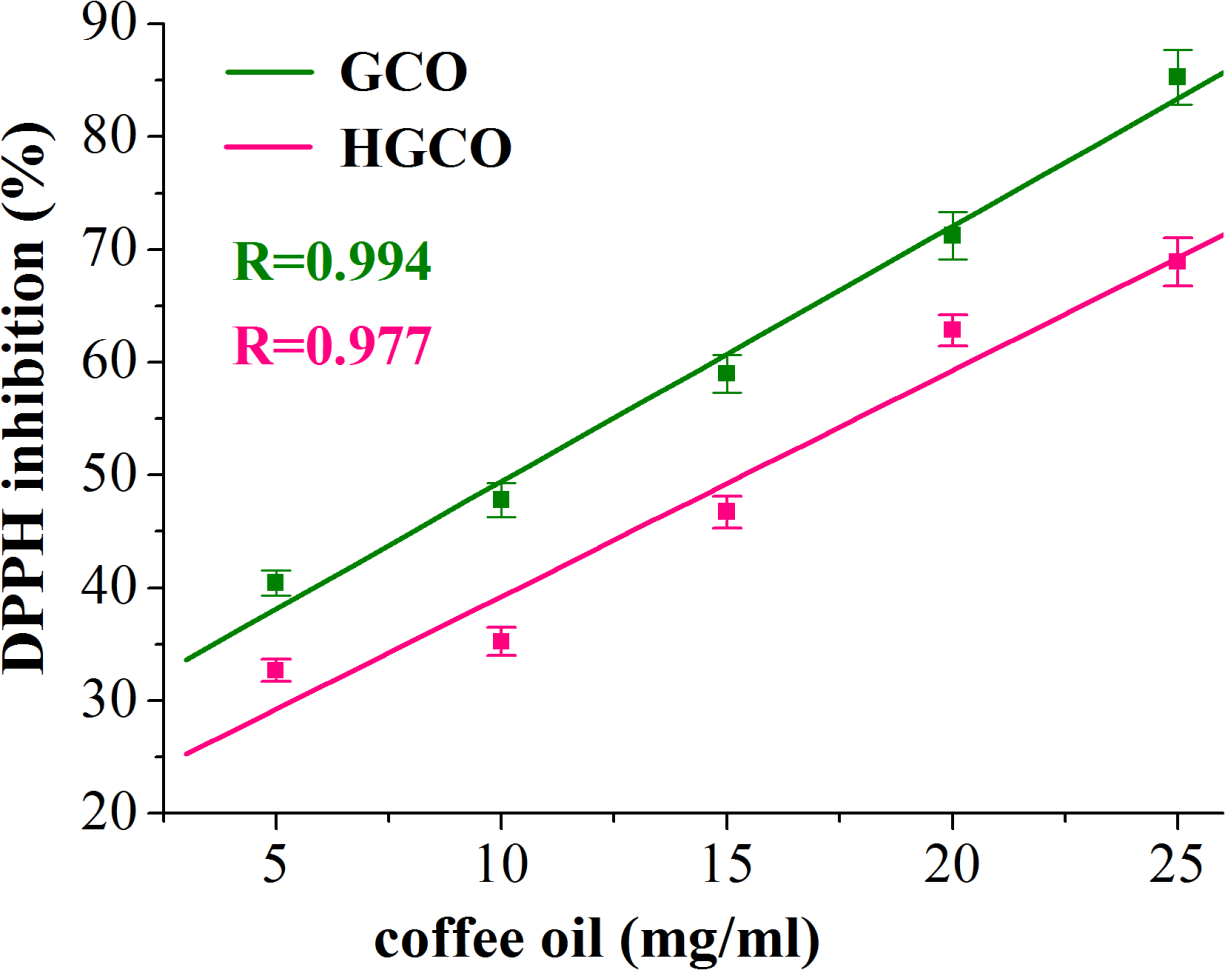
DPPH inhibition (%) *versus* concentration of GCO and HGCO.

**Fig 5:**
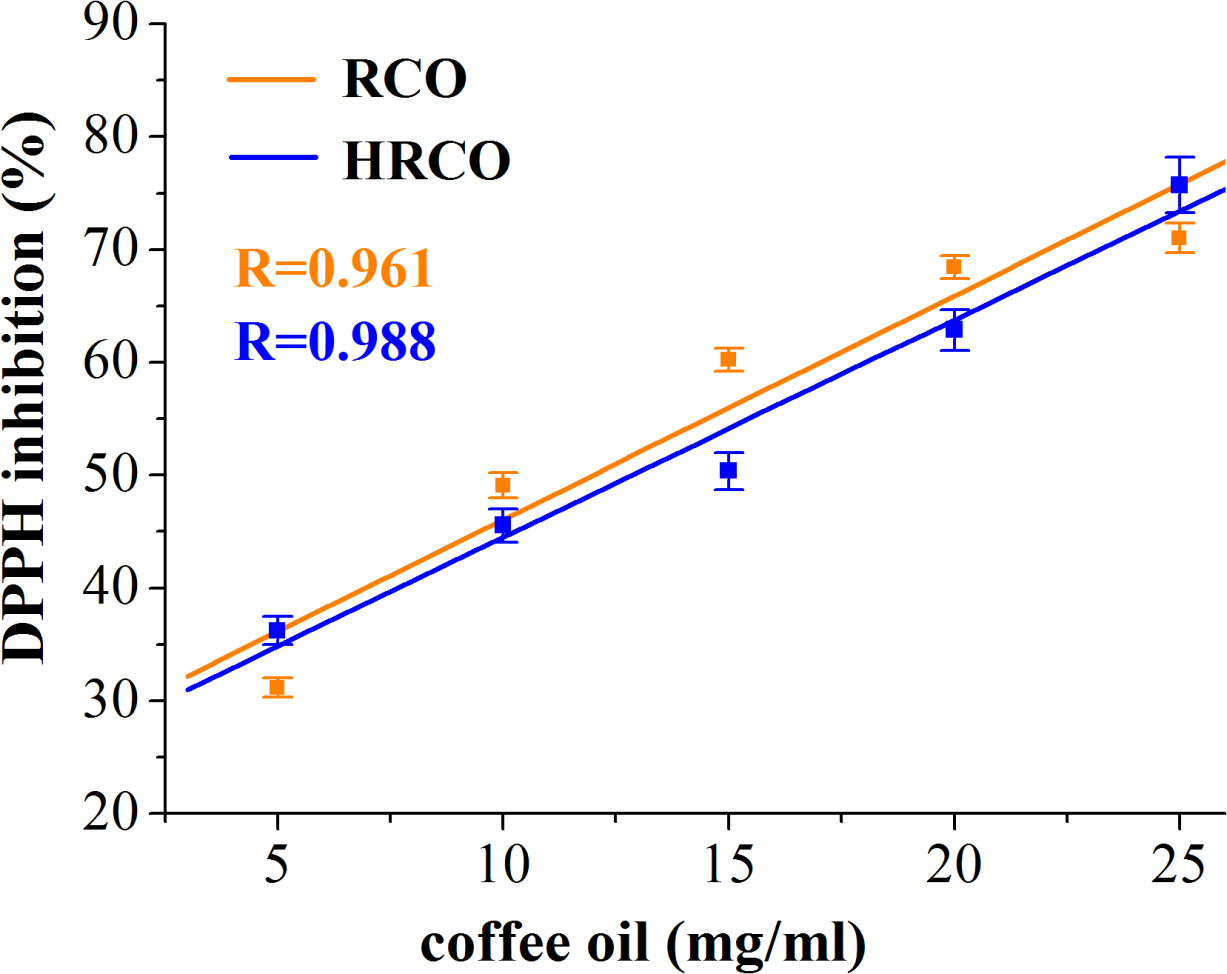
DPPH inhibition (%) *versus* concentration of RCO and HRCO.

**Fig 6:**
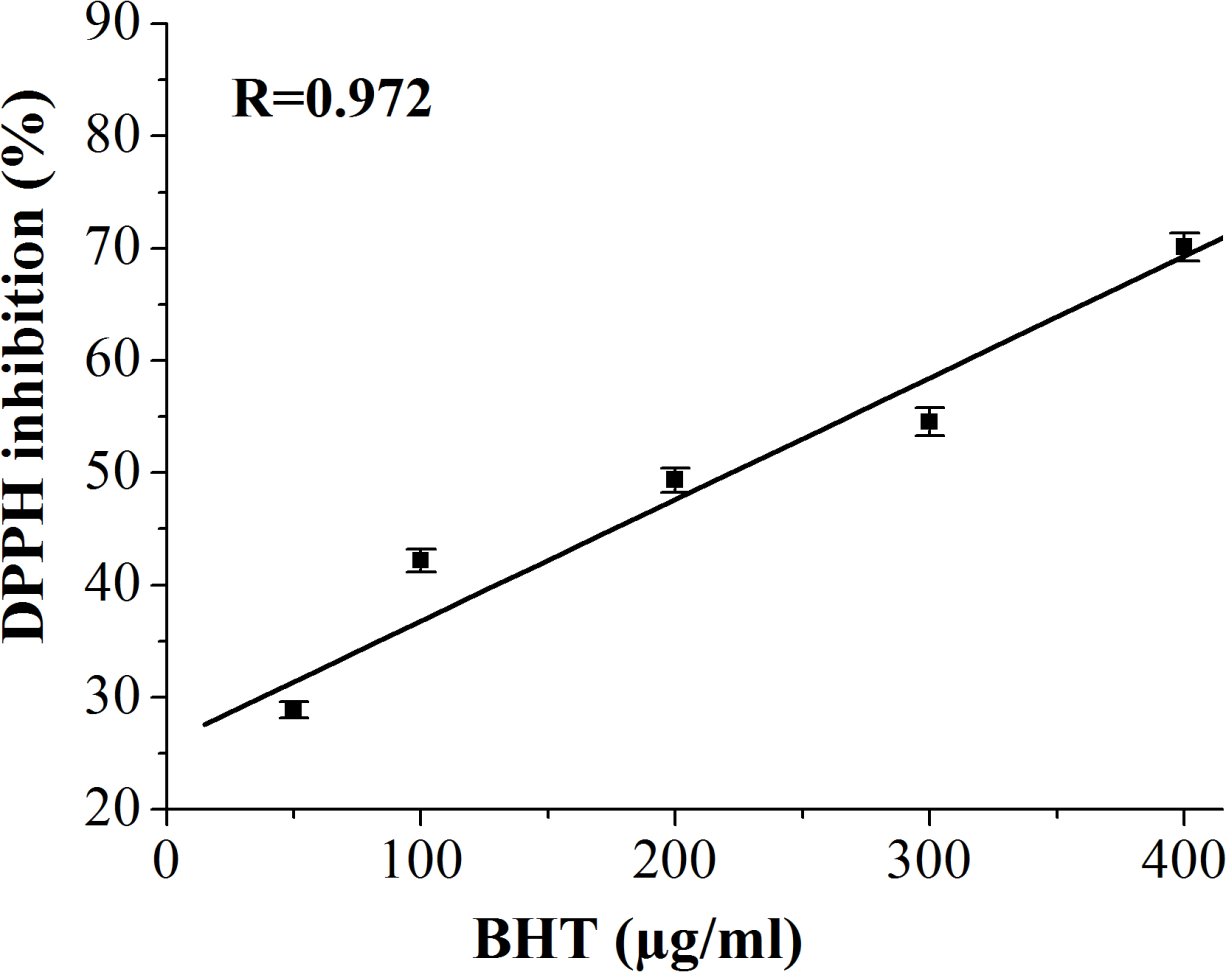
DPPH inhibition (%) *versus* concentration of BHT.

In this study, DPPH radical-scavenging assay was used for assessing the antioxidant activity of GCO and RCO and, in the same time, to investigate the oxidative stability of these oils in response to the high-temperature heat treatment. Thus, by this method the efficiency and the stability of natural antioxidants in crude and heated coffee oils were investigated. The results regarding the radical-scavenging activity, quantified by percentage of DPPH inhibition, related to the concentration of crude and heated coffee oil (using toluene as solvent) are depicted in Figs 4 and 5. In Fig 6 the antioxidant effectiveness of various concentrations of BHT is shown.

The IC50 values that express the concentration of sample required to scavenge 50% of DPPH radicals are shown in Table 4. Also, is pointed out the statistical significance of the differences recorded in the DPPH free radical scavenging ability of the investigated samples.

**Table 4:** The IC50 values of BHT and coffee oils.

Sample	IC50
BHT	219.67±10.43^a^ μg/ml
GCO	10.25±0.30^b^ mg/ml
HGCO	15.38±0.72^d^ mg/ml
RCO	11.99±0.51^c^ mg/ml
HRCO	12.86±0.79^c^ mg/ml

IC50 values sharing different superscripts (a-d) are significantly different from each other (one-way ANOVA, p<0.05).

Our results revealed a high correlation (correlation coefficient R>0.96) between the percentage of DPPH inhibition *versus* oil sample concentration. Thus, the radical scavenging ability of crude and heated coffee oils was concentration dependent. As it is shown in Table 4, the IC50 value of GCO was lower than of RCO. The recorded differences in the IC50 values of crude coffee oils were statistically significant different (one-way ANOVA, p<0.05). The IC50 values of both crude coffee oils were lower than those recorded for heated coffee oils, Table 4. The high temperature heating induced statistically significant differences in the IC50 values of GCO and HGCO (p<0.05) while there were no statistically significant changes (p>0.05) in the IC50 values of RCO and HRCO. The value of IC50 is inversely proportional to the scavenging activity of coffee oils. Hence, in heated coffee oil more concentration of antioxidant in the sample is required to inhibit the free radicals formed. The antioxidant activity of coffee oil samples was much lower than that of the synthetic BHT used as standard Table 4. Thus, the one-way ANOVA test revealed statistically significant differences (p<0.05) between IC50 values of BHT and both crude and heated coffee oil samples. The radical-scavenging activity of crude coffee oil, at different oil concentrations, was found to be better in GCO compared to RCO. The antioxidant activity of GCO and RCO is related to their content of natural antioxidants like sterols, phosphatides, tocopherols and diterpenes [5,8]. These results clearly show that the presence of antioxidant compounds in crude coffee oils decreased the absorbance of DPPH and increased the percentage of inhibition proportionally to the oil concentration. As it was reported by Speer and Kölling-Speer [8], the roasting diminishes the content of antioxidant compounds such as α-, β-tocopherol, and total tocopherols content. This finding could explain the lower radical-scavenging activity recorded for RCO than for GCO. As regards the diterpenes, there is no consensus in the literature data regarding the decrease of kahweol and cafestol contents during coffee beans roasting, but it has been reported that dehydrocafestol and dehydrokahweol are generated by dehydration of diterpenes under heat exposure [48]. Regarding the heat-treated oil samples, it supposes that in the in early stages of lipids oxidation the changes of the DPPH absorbance are due to the consumption of free radical scavengers while, during more advanced lipids oxidation, the formation of oxidized lipid compounds, able to react with DPPH radicals, is responsible for the changes recorded in DPPH absorbance [22, 23].

The free radicals generated in the heating time firstly reacted with antioxidant compounds from coffee oil samples and the remaining antioxidants reacted with DPPH. When the oil samples are depleted in antioxidant compounds, the free radicals formed through lipids oxidation reacted with DPPH and induced an increase in the percentage of the DPPH inhibition. Thus, the changes in the absorbance of DPPH could be used to determine the degree of lipid oxidation and antioxidant activity in lipid systems [22,49].

In the heat treated coffee oil samples the values of the DPPH inhibition do not reflect only the antioxidant potential of the remaining free radical scavengers but also the prooxidant properties of the oxidation products. Our findings are consistent with those reported by Yeo *et al*. [23] related to the fact that, once lipid oxidation progresses, the oxidized lipid compounds rather than the free radical scavengers play a more significant role in decreasing the absorbance of DPPH and consequently, in increasing of the DPPH radicals inhibition. The DPPH inhibition in HGCO revealed significant decreases as against GCO, but after 1 h of heat exposure there were no recorded changes in the pattern of DPPH inhibition *versus* coffee oil concentration, Fig 4. According to Lee *et al*. [49], vegetable oils containing many free radicals scavenging compounds require a relatively long oxidation time for changing the patterns of the DPPH absorbance. For HRCO, the free radicals generated in response to heating reacted with DPPH and led to an increase in the percentage of the DPPH inhibition. Thus, there were no important changes in the profiles of the DPPH inhibition *versus* RCO and HRCO concentrations (Fig 5). The results of our study reveal that the antioxidant activity of green and roasted coffee oil was different in response to the high-temperature heating, contributed by different antioxidant compounds with potential to scavenge the free radicals as well as by lipids oxidation products such as alkoxyl (RO∙), peroxyl (ROO∙), and alkyl radicals (R∙) generated in response to thermal stress.

As it was mentioned by Lee *et al*. [49], the DPPH method is not a measure of the amount of oxidation products but is closely related to the concentration of hydrogen donating antioxidants or generated lipid radicals by thermo-oxidative processes. Therefore, the changes recorded in the percentage of DPPH inhibition in response to free radicals formation in oxidized oils could be an indicator to predict the stability of oils undergoing thermal stress. These findings provide important information about the stability of antioxidant compounds of coffee oils investigated through DPPH method.

### Assessing the antimicrobial activity of coffee oil

The antimicrobial activity of investigated coffee oil samples and standard antibiotics used as control are shown in Table 5. The statistical significance of the differences in the antimicrobial activity exhibited by coffee oil samples and the standard antibiotics against the tested bacteria was appreciated on the basis of one-way ANOVA test.

**Table 5:** Antimicrobial activity of coffee oil and standard antibiotics.

	Microorganisms				
	*E*. *coli*	*E*. *faecalis*	*Salmonella*	*S*. *flexneri*	*S*. *aureus*
**Oil/Control**	**Diameter after 6 h of incubation (mm)**				
Cefotaxime	17±0.62^a^ (I)	–	–	–	–
Ampicillin	–	17±0.70^a^ (I)	–	–	25±1.05^a^ (S)
GCO	17±0.66^a^ (I)	16±0.60^a^ (I)	–	–	13±0.60^b^ (R)
HGCO	16±0.56^a^ (I)	8.5±0.36^b^ (R)	–	–	8±0.36^d^ (R)
RCO	8.5±0.30^c^ (R)	16±0.70^a^ (I)	–	–	10±0.46^c^ (R)
HRCO	10±0.46^b^ (R)	10±0.40^b^ (R)	–	–	8±0.40^d^ (R)
**Oil/Control**	**Diameter after 18 h of incubation (mm)**				
Cefotaxime	0 (R)	–	–	–	–
Ampicillin	–	0 (R)	25±1.01^a^ (S)	24±0.90^a^ (S)	25±1.05^a^ (S)
GCO	0 (R)	0 (R)	12±0.50^b^ (R)	8±0.30^b^ (R)	10±0.46^b^ (R)
HGCO	0 (R)	0 (R)	10±0.40^c^ (R)	0^c^ (R)	8±0.36^c^ (R)
RCO	0 (R)	0 (R)	0^d^ (R)	0^c^ (R)	8±0.30^c^ (R)
HRCO	0 (R)	0 (R)	0^d^ (R)	0^c^ (R)	8±0.40^c^ (R)

S: sensitive–exhibited antimicrobial activity (D>16 mm to Ampicillin); I: intermediate antimicrobial activity (D = 14–16 mm to Ampicillin; D = 15–22 mm to Cefotaxime); R: resistant—absent antimicrobial activity (D≤14 mm to Cefotaxime; D≤13 mm to Ampicillin); (–): bacterial culture shows no growth.

Values within the same column sharing different superscripts (a-d) are significantly different from each other (one-way ANOVA, p<0.05).

The effectiveness of coffee oil against Gram-positive bacteria (*E*. *faecalis*, *S*. *aureus)* and Gram- negative bacteria (*Salmonella*, *S*. *flexneri*, *E*. *coli)* was determined by the measuring of the inhibition zone diameter (D) and classified as sensitive, intermediate, resistant and the bacterial culture shows no growth, according to the clinical and the laboratory standards used [33]. After 6 h of incubation the measurable inhibition zone of *E*. *faecalis*, *S*. *aureus* and *E*. *coli* exhibited a low diameter against all the coffee oil samples compared to the control. There was no growth of *Salmonella* and *S*. *flexneri* strains recorded in the first 6 h of incubation. GCO and HGCO exhibited a similar antibacterial activity to the control against *E*. *coli* (D = 17 mm for GCO, respectively D = 16 mm for HGCO). Thus, there were not registered statistically significant differences between the control, GCO and HGCO (one-way ANOVA, p>0.05), Table 5. After 6 h of incubation GCO displayed the strongest antimicrobial activity against E. coli, followed by HGCO>RCO>HRCO. The results of one-way ANOVA analysis revealed that there were found statistically significant differences (p<0.05) between antimicrobial activity of GCO, RCO and HRCO. At the same time GCO and RCO showed a close antibacterial activity with the control against *E*. *faecalis* (D = 16 mm for both coffee oil samples). Also, GCO demonstrated the most powerful antimicrobial activity against *E*. *faecalis* followed by RCO>HGCO>HRCO. A statistically significant decrease (p<0.05) of antimicrobial activity of coffee oils was noticed after their heating. *S*. *aureus* was resistant against all investigated coffee oils after 6 h of incubation, as can be seen on the basis of the inhibition zone diameters, Table 5. The antimicrobial activity of all investigated coffee oils was statistically significant different to the control (p<0.05). After 18 h of incubation it was observed that the most tested bacterial species did not reveal visible and measurable inhibition zones in response to any coffee oil addition, except *Salmonella* (D = 10 mm for HGCO and D = 12 mm for GCO), *S*. *flexneri* (D = 8 mm for GCO) and *S*. *aureus* (D = 10 mm for GCO, RCO and HGCO, respectively D = 8 mm for HRCO). These species showed low inhibition zones in which appeared resistant colonies (D≤13 mm). These results suggest that both Gram-positive and Gram-negative bacteria species are not sensitive to the chemical compounds of coffee oils. After 18 h of incubation, the statistical analysis results highlighted that the investigated coffee oils revealed statistically insignificant antimicrobial activity (p>0.05) against all microbial strains tested compared to the control.

With respect to the tested bacterial species, it was found that the inhibition zone diameter decreased in the order *E*. *coli* > *E*. *faecalis* > *S*. *aureus* for the same coffee oil sample after 6 h of incubation and in the order *Salmonella* > *S*. *flexneri* > *S*. *aureus* after 18 h.

Our results demonstrate that GCO was found to be the most effective among investigated coffee oils against Gram-positive and Gram-negative tested bacteria. Nevertheless, GCO showed lower antimicrobial activity than the controls.

The results of antimicrobial assay demonstrate that coffee oils exhibited inhibitory activity against *E*. *coli* and *E*. *faecalis* only in the first 6 h of incubation. The variation recorded in the effectiveness of crude and heated oils against the tested bacteria is due to the thermal treatment.

The antimicrobial activity exhibited by coffee oil samples after 6 h of incubation could be associated with some compounds found in unsaponifiables matter of coffee oil, like diterpenes and tocopherols.

As it was reported by Anese *et al*. [12], the lipid fraction of coffee oil is relatively stable in response to the heat treatment. Thus, although diterpenes and tocopherols are sensitive to heat treatment, they may still be found in the lipid fraction of roasted coffee beans [8]. This result suggests that, even after heating in our conditions, these compounds could still be present. The diterpenes found in coffee (cafestol and kahweol) could be responsible for the antimicrobial activity of coffee oil exhibited in this study. Nowadays, there are only a few studies related to the antimicrobial effects of coffee or of its isolated compounds. The results reported by Ambrosio *et al*. [50] have revealed the effect of kaurane diterpenes against some oral pathogens. Recent studies on this topic conducted by Wagemaker *et al*. [4] have reported that GCO and some cosmetic formulations with GCO did not show antimicrobial activity against some Gram-positive and Gram-negative bacteria. As a result, considering that the unsaponifiable matter represents a significant part of GCO and RCO, further studies must be designed to elucidate the biological activities of coffee oil compounds.

## Conclusion

This study provides a solid and consistent evidence for the changes induced by high-temperature heating in the oxidative stability, antioxidant and antimicrobial properties of crude coffee oils. ATR-FTIR spectroscopy analysis highlighted that, there were no recorded significant differences (one-way ANOVA, p>0.05) in the oxidative status of GCO and RCO. The heat exposure of coffee oils induced statistically significant spectral changes in the regions 3100–3600 cm^–1^, 2800–3050 cm^–1^ and 1680–1780 cm^–1^ proved by the absorbance ratios RI-IV. These alterations were assigned to primary and secondary oxidation processes of the lipid fraction.

The IC50 value of GCO was significantly lower than of RCO (p<0.05). The IC50 values of crude coffee oils were lower than those of heated samples. The differences in the IC50 values of GCO and HGCO were statistically significant (p<0.05), while those between RCO and HRCO were not showed statistically significance (p>0.05). The antioxidant activity of coffee oils was contributed by different antioxidant compounds and lipids oxidation products generated in response to thermal stress. In the heated oils the DPPH inhibition reflects both the antioxidant potential of free radical scavengers and the prooxidant properties of oxidation products.

The inhibitory potential of crude coffee oils in the first 6 h of incubation was not statistically significant different related to the control (p>0.05) against *E*. *coli* and *E*. *faecalis* while the differences recorded for HGCO and HRCO were significantly different related to the control (p<0.05). The inhibitory effect of all coffee oils was statistically significant different (p<0.05) compared to the control against *S*. *aureus*. After 18 h the antibacterial activity of control was significantly higher (p<0.05) than of all coffee oils against the tested bacterial strains. GCO exhibited the highest antimicrobial potential against the tested Gram-positive and Gram-negative bacteria. Despite of the fact that some alterations are induced in the investigated properties by heat exposure, the coffee oil still possess an excellent potential to be used as ingredient in food industry.

## Abbreviations

GCO: green coffee oil
RCO: roasted coffee oil
HGCO: heated green coffee oil
HRCO: heated roasted coffee oil
ATR-FTIR spectroscopy: Attenuated Total Reflectance–Fourier Transform Infrared spectroscopy
A: absorbance
RI: absorbance ratio A 3009 cm^−1^/A 2922 cm^−1^
RII: absorbance ratio A 3009 cm^−1^/A 2853 cm^−1^
RIII: absorbance ratio A 3009 cm^−1^/A 1744 cm^−1^
RIV: absorbance ratio A 1744 cm^−1^/A 2922 cm^−1^
D: inhibition zone diameter
DPPH: 2,2-diphenyl-1-picrylhydrazyl
SD: standard deviation
p: probability
One-way ANOVA: One-way analysis of variance
R: correlation coefficient

## References

[1] Oliveira AL, Cruz PM, Eberlin MN, Cabral FA. Brazilian roasted coffee oil obtained by mechanical expelling: compositional analysis by GC-MS. Ciencia e Tecnologia de Alimentos. 2011; 25(4): 677–682.

[2] Calligaris S, Munari M, Arrighetti G, Barba L. Insights into the physicochemical properties of coffee oil. European Journal of Lipid Science and Technology. 2009; 111(12): 1270–1277.

[3] Wagemaker TAL, Carvalho CRL, Maia NB, Guerreiro Filho O. Sun protection factor, content and composition of lipid fraction of green coffee beans. Industrial Crops and Products. 2011; 33(2): 469–473.

[4] Wagemaker TAL, Fernandes AS, Campos PM, Rodrigues LM, Rijo P. Evaluation of antioxidant and antimicrobial activities of green coffee oil in cosmetic formulations. Biomedical and Biopharmaceutical Research. 2012; 2 (9): 207–214.

[5] Ferrari M, Ravera F, de Angelis E, Suggiliverani F, Navarini L. Interfacial properties of coffee oil. Colloids and Surfaces A: Physicochemical and Engineering Aspects. 2010; 365: 79–82.

[6] Gichimu BM, Gichuru EK, Mamati GE, Nyende AB. Biochemical composition within *Coffee croatia* cv. Ruiru 11 and its relationship with cup quality. Journal of Food Research, 2014; 3(3): 31–44.

[7] Tsukui A, Santos HM Júnior, Oigman SS, de Souza ROMA, Bizzo HR, Rezende CM. Microwave-assisted extraction of green coffee oil and quantification of diterpenes by HPLC. Food Chemistry. 2014; 164: 266–271. 10.1016/j.foodchem.2014.05.039 24996333

[8] Speer K, Kölling-Speer I. The lipid fraction of the coffee bean. Brazilian Journal of Plant Physiology. 2006; 18(1): 201–216.

[9] Cavin C, Holzhaeuser D, Scharf G, Constable A, Hubber WW. Cafestol and kahweol, two coffee specific diterpenes with anticarcinogenic activity. Food and Chemical Toxicology. 2002; 40(8): 1155–1158. 12067578 10.1016/s0278-6915(02)00029-7

[10] Hashim L, Chaveron H. Use of methylpyrazine ratios to monitor the coffee roasting. Food Research International. 1996; 28(6): 619–623.

[11] Czerny M, Grosch W. Potent odorants of raw Arabica coffee. Their changes during roasting. Journal of Agricultural and Food Chemistry. 2000; 48(3): 868–872. 10725165 10.1021/jf990609n

[12] Anese M, de Pilli T, Massini R, Lerici CR. Oxidative stability of the lipid fraction in roasted coffee. Italian Journal of Food Science. 2000; 12(4): 457–462.

[13] Vila MA, Andeuza S, Paz de Pena M, Cid C. Fatty acid evolution during the storage of ground, roasted coffees. Journal of the American Oil Chemists' Society. 2005; 82(9): 639–646.

[14] Frascareli EC, Silva VM, Tonon RV, Hubinger MD. Effect of process conditions on the microencapsulation of coffee oil by spray drying. Food and Bioproducts Processing. 2012; 90(3): 413–424.

[15] Alvarez AMR, Rodriguez MLG. Lipids in pharmaceutical and cosmetic preparations. Grasas Aceites. 2000; 51(1–2): 74–96.

[16] Del Carmen Velasquez Pereda M, De Campos Dieamant G, Eberlin S, Nogueira C, Colombi D, Di Stasi LC, et al Effect of green Coffea arabica L. seed oil on extracellular matrix components and water-channel expression in in vitro and ex vivo human skin models. Journal of Cosmetic Dermatology. 2009; 8(1): 56–62. 10.1111/j.1473-2165.2009.00425.x 19250168

[17] de Oliveira PMA, de Almeida RH, de Oliveira NA, Bostyn S, Gonçalves CB, de Oliveira AL. Enrichment of diterpenes in green coffee oil using supercritical fluid extraction–Characterization and comparison with green coffee oil from pressing. The Journal of Supercritical Fluids. 2014; 95: 137–145.

[18] Herrera FC, Santos JA, Otero A, García-López ML. Occurrence of foodborne pathogenic bacteria in retail prepackaged portions of marine fish in Spain. Journal of Applied Microbiology. 2006; 100: 527–536. 16478492 10.1111/j.1365-2672.2005.02848.x

[19] Gutiérrez D, Delgado S, Vázquez-Sánchez D, Martínez B, Cabo ML, Rodríguez A, et al Incidence of Staphylococcus aureus and analysis of associated bacterial communities on food industry surfaces. Applied and Environmental Microbiology. 2012; 78(24): 8547–8554. 10.1128/AEM.02045-12 23023749 PMC3502933

[20] Shijie W, Duan H, Zhang W, Li JW. Analysis of bacterial foodborne disease outbreaks in China between 1994 and 2005. FEMS Immunology & Medical Microbiology. 2007; 51(1): 8–13. 17666075 10.1111/j.1574-695X.2007.00305.x

[21] Guillen MD, Cabo N, Ibargoitia ML, Ruiz A. Study of both sunflower oil and its headspace throughout the oxidation process. Occurrence in the headspace of toxic oxygenated aldehydes. Journal of Agricultural and Food Chemistry. 2005; 53: 1093–1101. 15713025 10.1021/jf0489062

[22] Yeo JD, Jeong MK, Lee JH. Application of DPPH absorbance method to monitor the degree of oxidation in thermally-oxidized oil model system with antioxidants. Food Science and Biotechnology. 2010; 19(1): 253–256.

[23] Yeo JD, Jeong MK, Lee JH. Correlation of antioxidant content and absorbance changes of DPPH during lipid oxidation. Food Science and Biotechnology. 2012; 21(1): 199–203.

[24] Jeong MK, Yeo JD, Jang EY, Kim MJ, Lee JH. Aldehydes from oxidized lipids can react with 2,2-Diphenyl-1-Picrylhydrazyl (DPPH) free radicals in isooctane systems. Journal of the American Oil Chemists' Society. 2012; 89: 1831–1838.

[25] Guillen MD, Cabo N. Fourier transform infrared spectra data versus peroxide and anisidine values to determine oxidative stability of edible oils. Food Chemistry. 2002; 77: 503–510.

[26] Vlachos N, Skopelitis Y, Psaroudaki M, Konstantinidou V, Chatzilazarou A, Tegou E. Applications of Fourier transform–infrared spectroscopy to edible oils. Analytica Chimica Acta. 2006; 573–574: 459–465. 17723561 10.1016/j.aca.2006.05.034

[27] Rohman A, Man YBC, Ismail A, Hashim P. Monitoring the oxidative stability of virgin coconut oil during oven test using chemical indexes and FTIR spectroscopy. International Food Research Journal. 2011; 18: 303–310.

[28] Guillen M, Cabo N. Relationships between the composition of edible oils and lard and the ratio of the absorbance of specific bands of their Fourier transform infrared spectra. Role of some bands of the fingerprint region. Journal of Agricultural and Food Chemistry. 1998; 46: 1788–1793.

[29] Moharam MA, Abbas LM. A study on the effect of microwave heating on the properties of edible oils using FTIR spectroscopy. African Journal of Microbiology Research. 2010; 4: 1921–1927.

[30] AOCS (American Oil Chemists’ Society). Official methods and recommended practices of the AOCS 5th edition, editor Firestone D., AOAC Press, Champaign Illinois, USA; 1998.

[31] Ramadan MF, Moersel JT. Screening of the antiradical action of vegetable oils. Journal of Food Composition and Analysis. 2006; 19(8): 838–842.

[32] CLSI: Clinical and Laboratory Standards Institute. Performance standards for antimicrobial susceptibility testing: Twenty First International Supplement M100-S21. Clinical and Laboratory Standards Institute, Wayne, PA; 2011.

[33] SAS Institute Inc SAS for Windows, Version 8.1 Edition. Cary, North Carolina: SAS Institute Inc.; 2000.

[34] Ravindranath R, Yousuf Ali Khan R, Obi Reddy T, Thirumala Rao SD, Reddy BR. Composition and characteristics of Indian coffee bean, spent grounds and oil. Journal of the Science of Food and Agriculture. 1972; 23(3): 307–310.

[35] Hartmann L, Lago RCA, Tango JS, Teixeira CG. The effect of unsaponifiable matter on the properties of coffee seed oil. Journal of the American Oil Chemists' Society. 1968; 45: 577–579.

[36] Lago RCA. Lipídios em grãos de café. Boletim CEPPA (Centro de Pesquisa e Processamento de Alimentos). 2001; 19(2): 319–341.

[37] Jović O, Smolić T, Jurišić Z, Meić Z, Hrenara T. Chemometric analysis of croatian extra virgin olive oils from central Dalmatia region. Croatica Chemica Acta. 2013, 86: 335–344.

[38] Van de Voort F, Ismail A, Sedman J, Emo G. Monitoring the oxidation of edible oils by FTIR spectroscopy. Journal of the American Oil Chemists' Society. 1994; 71: 243–253.

[39] Bach D, Miller IR. Attenuated total reflection (ATR) Fourier transform infrared spectroscopy of dimyristoyl phosphatidylserine–cholesterol mixtures. BBA Biomembranes. 2001; 1514: 318–326. 11557030 10.1016/s0005-2736(01)00388-1

[40] Toci AT, Neto VJMF, Torres AG, Farah A. Changes in triacylglycerols and free fatty acids composition during storage of roasted coffee. LWT–Food Science and Technology. 2013; 50(2): 581–590.

[41] Nicoli MC, Anese M, Manzocco L, Lerici CR. Antioxidant properties of coffee brews in relation to the roasting degree. LWT–Food Science and Technology. 1997; 30(3): 292–297.

[42] Caemmerer B, Kroh LW. Antioxidant activity of coffee brews. European Food Research and Technology. 2006; 223(4): 469–474.

[43] Amarowicz R. Antioxidant activity of Maillard reaction products. European Journal of Lipid Science and Technology. 2009; 111(2): 109–111.

[44] Martinez-Yusta M, Goicoechea E, Guillen MD. A review of thermo-oxidative degradation of food lipids studied by 1H NMR spectroscopy: influence of degradative conditions and food lipid nature. Comprehensive Reviews in Food Science and Food Safety. 2014; 13: 38–859.

[45] Osmund Falade A, Oboh G. Thermal oxidation induces lipid peroxidation and changes in the physicochemical properties and β-carotene content of arachis oil. International Journal of Food Science. 2015; vol. 2015, 7 pages, Article ID 806524, 10.1155/2015/806524 PMC4745487 26904665

[46] Yoshida H, Tatsumi M, Kajimoto G. Influence of fatty acids on the tocopherol stability in vegetable oils during microwave heating. Journal of the American Oil Chemists' Society. 1992; 69: 119–125.

[47] Moreno M, Olivares M, López A, Adelantado J, Reig B. Analytical evaluation of polyunsaturated fatty acids degradation during thermal oxidation of edible oils by Fourier transform infrared spectroscopy. Talanta. 1999; 50: 269–275. 18967717 10.1016/s0039-9140(99)00034-x

[48] Dias RCE, de Faria-Machado AF, Mercadante AZ, Bragagnolo N, de Toledo Benas M. Roasting process affects the profile of diterpenes in coffee. European Food Research and Technology. 2014; 239(6): 961–970.

[49] Lee JM, Chung H, Chang PS, Lee JH. Development of a method predicting the oxidative stability of edible oils using 2,2-diphenyl-1- picrylhydrazyl (DPPH). Food Chemistry. 2007; 103(2): 662–669.

[50] Ambrosio SR, Furtado NA, de Oliveira DC, da Costa FB, Martins CH, de Carvalho TC, et al Antimicrobial activity of kaurane diterpenes against oral pathogens. VZN–Section C–Verlag der Zeitschrift für Naturforschung. 2008; 63(5–6): 326–30. 10.1515/znc-2008-5-603 18669015

